# Assessment of the endocrine disrupting properties of bisphenol AF: a case study applying the European regulatory criteria and guidance

**DOI:** 10.1186/s12940-021-00731-0

**Published:** 2021-04-24

**Authors:** Laura Escrivá, Johanna Zilliacus, Ellen Hessel, Anna Beronius

**Affiliations:** 1 Faculty of Pharmacy, University of Valencia, Burjassot, Valencia, Spain; 2 Karolinska Institutet, Institute of Environmental Medicine, Stockholm, Sweden; 3 RIVM National Institute for Public Health and the Environment, Utrecht, The Netherlands

**Keywords:** Bisphenol AF, Endocrine disruptors, Risk assessment, Pesticides regulation, REACH, Weight of evidence evaluation

## Abstract

**Background:**

Scientific criteria to identify endocrine disruptors (ED) was recently implemented for plant protection products (PPP) and biocidal products (BP). A guidance document has been published by ECHA and EFSA in the context of ED criteria for PPPs and BPs.

**Methods:**

In the present work, a case study was performed on Bisphenol AF (BPAF) to explore the application of the EU criteria and EFSA/ECHA guidance document for the ED assessment of a non-pesticide chemical regulated under REACH. A data dossier was built by a systematic literature search (Web of Science, Pubmed, Embase; *n* = 511), title/abstract screening (*n* = 124) and full text examination (*n* = 88). All the information was extracted and systematically reported for 309 parameters (100 for adversity; 209 for endocrine activity). The reliability of studies was assessed (SciRAP tool).

**Results:**

Data were synthesized into 96 lines of evidence for adversity (*n* = 57), and endocrine activity (*n* = 39); and assessed by weight of evidence methodology. The initial analysis of the evidence indicated EATS-mediated adversity in mammals, therefore a mode of action (MoA) was postulated for both male and female adult exposure. Female MoA included estrogen receptor activation and altered steroidogenesis leading to ovarian dysfunction, altered estrous cycling and impaired female fertility. Male MoA was initiated by androgen receptor inhibition and altered steroidogenesis leading to dysfunction of male reproductive organs and impaired male fertility.

**Conclusions:**

The overall conclusion of the ED assessment indicated that BPAF meets the ED criteria for human health. The steps described in the ED guidance document were successfully completed, resulting in a thorough, structured and transparent identification of BPAF as an ED. Advantages and limitations of applying the ED criteria and guidance for a REACH chemical are discussed.

**Supplementary Information:**

The online version contains supplementary material available at 10.1186/s12940-021-00731-0.

## Background

An endocrine disruptor (ED) is an exogenous substance or mixture that alters function(s) of the endocrine system and consequently causes adverse health effects in an intact organism, or its progeny, or (sub) populations [1]. A heterogeneous group of chemicals, including pesticides, fungicides, plastics, plasticizers and heavy metals, have been observed to interact with the endocrine system. As a consequence, humans and animals are exposed to diverse mixtures of potential EDs from several matrices such as food and other consumer products [2]. EDs represent a special and challenging form of toxicity as their effects depend on both the level and timing of exposure, being especially critical during early development [1]. Scientific understanding of the health impacts of EDs has been growing in recent years and progressively raised awareness of ED related concerns [3]. EDs are, for example, an area of focus in the European Commission’s recently published Chemicals Strategy for Sustainability Towards a Toxic-Free Environment [4]. Nevertheless, to demonstrate that a given substance is an ED represents a huge challenge due to the complexity of the endocrine system in maintaining the homeostasis of all biological processes, as well as the multiple pathways and mechanisms involved [5].

In the last two decades, the European Commission initiated a strategy to develop a legislative framework for EDs, pursuing the harmonization of hazard-based criteria for EDs identification. The three elements required to identify an ED were described in line with the WHO definition [6] an ED substance has to show i) endocrine adverse health effects in individuals and/or their offspring, ii) endocrine activity and, iii) a plausible and clear-established link between the adverse effects and the endocrine activity [7, 8]. Scientific criteria to identify substances with ED properties have been recently implemented for plant protection products (PPP) [9], and biocidal products (BP) [10] applying from June and November 2018, respectively. The criteria dictate that all data relevant for ED assessment should be considered using systematic review methodology and weight of evidence (WoE) evaluation. A guidance document for the implementation of ED criteria pursuant to the PPP and BP regulations has been developed by the European Food Safety Authority (EFSA) and the European Chemicals Agency (ECHA). The recently published ECHA/EFSA guidance document intends to reduce subjectivity and conflicting procedures for determining ED properties by guiding applicants and assessors of the competent regulatory authorities, contributing to the harmonization between industry, authorities and academia with regard to ED assessment [7]. The ED guidance document describes a strategy to assess whether a substance meets the EU scientific criteria as an ED with regard to the risk to humans and other non-target organisms. Although the ED criteria cover all ED effects the ED guidance document mainly addresses estrogen, androgen, thyroid, steroidogenesis (EATS) modalities. For the EA modalities there is relatively good mechanistic understanding of how substance-induced perturbations to these modalities may lead to adverse effects in vivo*.* There are available standardized test guidelines for in vivo and in vitro testing [11], and there is broad scientific agreement on the interpretation of the effects observed on the investigated parameters [7]. However, it is still difficult to link in vivo effects measured in these tests to adverse effects in humans, especially for the thyroid and steroidogenesis modalities. A structured strategy describing the steps necessary to identify an ED substance is provided in the ED guidance document and briefly described as follows: 1) gathering all relevant information, including both regulatory toxicity tests and other relevant data from databases and the scientific literature; 2) evaluating relevance and reliability of the available data; 3) extracting and transparently reporting the information in a tabular form including all the parameters useful for the ED assessment, as well as data on systemic toxicity for both positive and negative results; 4) assembling and assessing the lines of evidence for endocrine activity and adversity considering all the available evidence (positive and negative) that have been assessed as relevant and reliable; 5) initial analysis of the evidence (assessment whether either EATS-mediated adversity or EATS endocrine activity has been sufficiently investigated and/or observed); 6) MoA analysis, if required; and 7) overall conclusion on the ED criteria.

Under the European REACH (Registration, Evaluation, Authorization and Restriction of Chemicals) regulation, substances having ED properties may fulfil the definition of Substances of Very High Concern (SVHC) as first step towards regulation and appropriate risk management. The use of SVHC is controlled by temporary authorizations conditioning its uses and strongly encouraging its substitution [5, 12]. This is also highlighted in the European Commission’s Chemicals Strategy [4].

The REACH regulation does not currently provide any specific ED criteria. However, the European Commission has communicated an intention for the development of a horizontal approach for ED identification across EU legislation built on the ED criteria established for PPPs and BPs [3].

**Fig. 1:**
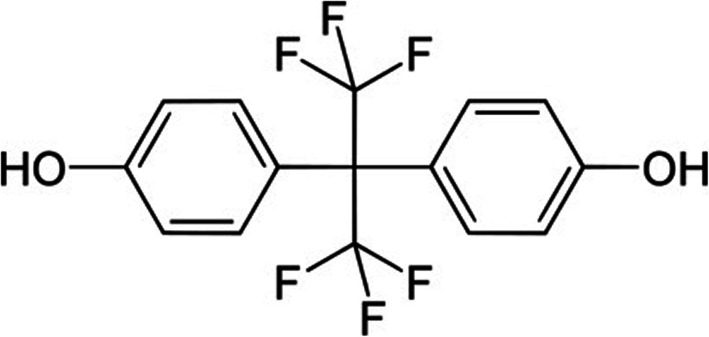
Bisphenol AF (BPAF) chemical structure; 4-[1,1,1,3,3,3-hexafluoro-2-(4-hydroxyphenyl)propan-2-yl]phenol; CAS number: 1478-61-1

Bisphenol AF (BPAF) [4-[1,1,1,3,3,3-hexafluoro-2-(4-hydroxyphenyl)propan-2-yl]phenol; Fig. 1] is a structural analog to Bisphenol A (BPA; CAS No. 80–05-7) where both methyl groups are replaced to trifluoromethyl groups [13]. BPAF is increasingly being used as one of the substitutes to BPA [14], as the use of BPA has been restricted in the EU [15, 16] due to its identification as an SVHC and ED chemical (for both the environment and human health) under REACH. In contrast to the extensive evaluation performed on the ED properties of BPA, most BPA analogues, including BPAF, are less well understood with respect to their potential toxicity [17, 18]. With the increasing exposure to BPA-substitutes it is imperative to determine whether the exposure to these compounds, especially during the embryonic and developmental period, results in similar ED effects as previously identified by BPA. An increasing number of recent studies have shown comparable effects on the endocrine system by BPA alternatives suggesting that BPA analogs can have equal and in some cases greater ED effects as BPA, highlighting the need for detailed toxicity studies on BPA analogs as potential safer alternatives to BPA [19, 20]. BPAF is manufactured and/or imported in the European economic area in 100–1000 t/ year and is indicated to be toxic to reproduction [21]. BPAF has shown activity as an agonist on estrogen receptors (ERs) in several in vitro assays, with indications that the estrogen activity might be greater than BPA, as well as anti-androgen activity comparable to BPA [19].

This case study explores the hypothesis that the EU criteria and ED guidance document for ED assessment of PPPs and BPs may be successfully applied in the assessment of ED properties of a non-pesticide compound. The aim was to investigate and illustrate, step-by-step, the application of the ED assessment process on BPAF as a model substance, to identify the scientific strengths and challenges in this process, as well as to evaluate its applicability in the evaluation of a REACH chemical. The conclusions regarding the ED properties of BPAF for human health, as well as insights regarding the ED assessment process for chemicals regulated under REACH are discussed.

## Materials and methods

BPAF was selected as a suitable and relevant model substance for this case study due to the amount of available data, the concern for human exposure and risk, and its similarities to BPA, a known ED.

### Systematic literature search

A systematic literature search of the scientific literature (academic studies) on BPAF was performed based on the principles of systematic review (SR) methodology [22].

The problem formulation (review question) and the PECO (Population, Exposure, Comparator, Outcome) statements were predefined, and the protocol for the study inclusion/exclusion criteria was established (Supplementary material, Tables S1, S2). The electronic databases Web of Science, Pubmed, and Embase were used for gathering all data by a single concept search strategy. Such a strategy is based only on the Exposure in the PECO, i.e. searching for the compound name, synonyms or other identifiers, such as CAS number. This generates a highly sensitive search aimed at capturing all available data on the compound under study. In contrast, a targeted search strategy may be applied if a single concept search retrieves a very large number of irrelevant hits and also includes the Outcome from the PECO, i.e. refining the search by using specific search terms for the endpoints of interest for the specific problem formulation. The output of the single concept search for BPAF did not retrieve excessively large number of hits, therefore, further refinement by running a targeted search was not required. The search included relevant search terms including CAS number, IUPAC name, and chemical name synonyms of BPAF (Supplementary material, Table S3). All databases were searched on the 15th February 2018, but Embase on 12th April 2019, and the retrieved studies were transferred into the electronic reference management software EndNote where reference duplicates were removed to obtain the preliminary dossier. In addition, searches were conducted in eChemPortal, in ToxCast (https://comptox.epa.gov/dashboard) and in the REACH registration dossier for BPAF available in the ECHA database (https://echa.europa.eu/es/registration-dossier/-/registered-dossier/23236/7/6/1).

### Screening and selection of the studies

In order to identify the relevant studies captured in the literature search (preliminary dossier), two selection steps were applied: i) title and abstract screening, and ii) full text examination. The retrieved studies were transferred from EndNote software to RAYYAN tool (https://rayyan.qcri.org/). Title and abstract screening was independently performed by two reviewers at different institutions and countries using the RAYYAN tool under ‘blind on’ mode. The screening and selection of studies was based on the predefined problem formulation, PECO statements and study inclusion/exclusion criteria (Supplementary material, Table S2). Studies meeting the inclusion criteria were kept for next screening step, while studies clearly not relevant to problem formulation or meeting the exclusion criteria were excluded. When exclusion could not be made based on the title/abstract, studies were kept for subsequent full text examination. Conflicts between the two reviewers regarding the inclusion or exclusion of studies were resolved by discussion. The included studies formed the title and abstract dossier (screening dossier) and were subjected in a second step to deep examination at full-text level performed by one reviewer. Studies considered eligible after full-text screening were included into the full text dossier (final dossier) and preliminary classified based on the nature of the data as: epidemiological, in silico, in vitro*,* in vivo/mammals, and in vivo/non-mammals.

### Extracting and reporting the information

All studies included in the final dossier were coded with ID numbers (Supplementary material Appendix A). All the information from the included studies was extracted and systematically reported using the supplementary excel-based tool from the ED guidance document document (appendix E - excel template for reporting the available information relevant for ED assessment (https://efsa.onlinelibrary.wiley.com/doi/full/10.2903/j.efsa.2018.5311). As recommended in the ED guidance document, relevant endocrine-related parameters, as well as general toxicity endpoints were included and both positive and negative results were reported. For assessment of endocrine activity, in vitro and in vivo mechanistic data from both mammals and non-mammals were extracted based on the consideration that mechanisms for endocrine activity may be relevant across species. Only data from studies in mammals were extracted for the assessment of adversity, because of limited relevance of non-mammalian data to human health, and data did not contain non-mammalian assays of level 4 and 5 that could provide insights for adversity in humans according to the OECD guideline [11].

To collect all data, each parameter (i.e. an effect/endpoint/outcome measured in a study) was extracted in one single row in the excel. Accordingly, each row reports the changes observed in a certain parameter within a specific study. Note that multiple effects could have been investigated in a single study; in this case it was assigned a row for each single effect in the template, therefore the same study appeared several times. All relevant information for each single parameter was extracted following the template indications (eg. type of toxicity, study principle, species/strain or in vitro model, animals/sex/group, substance purity, route of administration, method of administration, tested doses, duration of exposure, generation/life stage, sex effect dose, lowest effect dose, effect type, effect target, effect classification, effect description, effect determination, effect direction). The excel template includes a function that allows the reorganization of data into a data matrix with all effects observed from one study shown on one row, which facilitates summarizing the information.

### Evaluating reliability

Individual in vivo and in vitro studies included in the dossier were evaluated for reliability by one reviewer using the web-based Science in Risk Assessment and Policy (SciRAP) tool (http://www.scirap.org). The SciRAP study evaluation tool is based on pre-defined criteria for reliability, including reporting and methodological quality [23]. Evaluation of study reliability using SciRAP consists of 23 reporting quality criteria and 15 methodological quality criteria for in vitro studies; and 30 reporting quality criteria and 18 methodological quality criteria for in vivo studies. The output for each assessed study is provided as an excel-file containing a colour profile (qualitative evaluation) and score from 0 to 100 (quantitative evaluation) for study reliability. In this study, reliability categorization was based on the SciRAP score for methodological quality, distinguishing three categories: i) reliable (score ≥ 75), ii) partially reliable (score 60–74), and iii) not reliable (score < 60). When a study contained both in vitro and in vivo information two independent SciRAP evaluations were performed. WoE evaluation of lines of evidence (see section 2.6) was based on data from reliable and partially reliable studies, considering the information rated as not reliable not determinant for the overall assessment.

### Assembling lines of evidence

After re-organizing the extracted parameters into the data matrix in the excel template, parameters were further grouped into lines of evidence, i.e. sets of relevant information (parameters) grouped together to assess a hypothesis [24]. The term ‘parameter’ includes a single effect evaluated in one study, while ‘lines of evidence’ are groups of similar effects (parameters) evaluated in different studies that may lead to a conclusion. In this case, lines of evidence were, for example, data on hormone levels, gene expression, organ-specific effects. Lines of evidence were organized into two groups as providing evidence for adversity or endocrine activity, respectively, in accordance with the guidance recommendations, and they were further grouped based on the nature of the data as shown in Table 1. Mammal and non-mammal data were evaluated separately. Although the assessment was focused on ED effects, lines of evidence for general toxicity were also reported and evaluated since, according to the ED guidance document, all the information from the dossier including additional data (e.g. systemic general toxicity or target organ effects) should be extracted to contextualize the presence or absence of an adverse effect potentially linked to an endocrine activity.

**Table 1:** Organization of lines of evidence for adversity and endocrine activity

Adversity	Endocrine activity
EATS-mediated	In vitro mechanistic
EATS-sensitive but not diagnostic	In vivo mechanistic (mammals)
General toxicity	In vivo mechanistic (non-mammals)

### Assessing, integrating and reporting lines of evidence

The available lines of evidence were assessed by applying WoE evaluation based on examples from the ED guidance document as well as a guidance for WoE evaluation from the European Commission Scientific Committee on Health, Environmental and Emerging Risks [25]. In the resulting approach, each line of evidence was evaluated based on the quality (as assessed using the SciRAP tool), as well as consistency among studies and species. Principles for categorizing the WoE of each line of evidence as “strong”, “moderate” or “weak” were developed specifically for this study (Table 2). Note that these describe the evidence for effects that have been observed. Lines of evidence where no effects were apparent were labelled as “no evidence for effect” and no conclusions on weight of evidence were made. It is also important to point out that “no evidence for effect” should not be interpreted as evidence for the absence of effect.

**Table 2:** WoE categories for lines of evidence assessment

Category	Principles for categorization
**Strong**	■Effects were observed in one or more studies of high reliability; there are no conflicting results.
**Moderate**	■Effects were observed in one study of partial reliability, or ■effects were observed in two or more studies of partial reliability; there are no conflicting results, or ■effects were observed in one or more studies of high or partial reliability but with conflicting results, i.e., no or opposite effects were observed in other studies. However, conflicts of results can be explained by differences in study design, for example different exposure periods, doses or animal species or cell models.
**Weak**	■Effects were observed in one or more studies of high or partial reliability but with conflicting results, i.e., no or opposite effects were observed in other studies. Conflicts of results cannot be explained by differences in study design, for example different exposure periods, doses or animal species or cell models, or ■effects were only observed in one or more studies of low reliability.

The lines of evidence were then integrated for an overall evaluation of whether the data set was sufficient to support a conclusion on adversity and/or endocrine activity for EAS- and T-modalities, respectively as described in the ED guidance document [7].

### Initial analysis of the evidence

The available data in the BPAF dossier were evaluated with regard to whether EATS-mediated adversity and EATS-related endocrine activity had been sufficiently investigated, as well as if adversity/endocrine activity had been observed. The ED guidance document provides a description of what is considered a sufficient data set in the context of assessing ED properties of PPPs and BPs, which is based on the endpoints measured in standardized OECD studies included in the OECD Conceptual Framework for Testing and Assessment of Endocrine Disrupters [11]. Six scenarios are described in the ED guidance document (section 3.4.4). Briefly, if adversity has been sufficiently investigated two scenarios are possible: scenario 1a (no adversity is observed based on EATS-mediated parameters, therefore ED criteria are not met), and scenario 1b (adversity is observed based on EATS-mediated parameters, therefore mode of action (MoA) analysis should be performed). On the contrary, if adversity has not been sufficiently investigated four scenarios are possible: scenario 2b (adversity is observed based on EATS-mediated parameters); scenario 2a (i) (no EATS-mediated adversity is observed but endocrine activity is observed); scenario 2a (ii) (no EATS-mediated adversity nor endocrine activity is observed and endocrine activity has been sufficiently investigated); or scenario 2a (iii) no EATS-mediated adversity nor endocrine activity is observed but endocrine activity has not been sufficiently investigated. In scenarios 1a and 2a(ii), the conclusion is that the ED criteria are not met. In scenario 2a(iii) further data need to be generated. In scenarios 1b, 2a(i) and 2b MoA analysis should be the next step of the assessment.

### Mode of action analysis

A MoA is a sequence of measurable events at molecular, cellular, organ and organism levels that link a molecular initiating event (MIE), to an adverse outcome (AO) though intermediate key events (KEs). In the context of ED assessment, the MoA analysis consists of two steps: 1) postulating a MoA, and 2) evaluating the MoA by establishing the biologically plausible link between endocrine activity and adverse effect [7].

The MoA analysis was based on WoE approach and adverse outcome pathways (AOP) methodology [25]. For MoA postulation, the adverse effects that showed strong WoE were initially selected as potential AOs. The information in the lines of evidence biologically connected to these AOs was organized into different levels of biological organization to hypothesize potential KEs. Two preliminary MoAs were postulated defining the chain of KEs from the molecular/cellular level to the AOs at individual level. An overall conclusion of the ED properties of BPAF was reported based on the postulated MoA.

## Results

### Systematic literature search, screening and selection of studies

The systematic literature search performed by a single concept approach retrieved 446 (Web of Science), 168 (Pubmed) and 225 (Embase) items (Supplementary material, Table S4).

After duplicates removal 511 studies were included in the preliminary dossier, and a refined targeted search was not considered necessary. Title/abstract screening of the 511 studies based on the PECO statements and eligibility criteria was then performed leading to 128 and 120 included items for each reviewer, respectively. After moving to ‘blind off’ mode in RAYYAN 7 items in conflict were discussed until agreement, concluding with 124 (24%) studies included in the screening dossier, while 387 studies not meeting the eligibility criteria were excluded for the next screening step. The main reasons for exclusion at title/abstract screening were: irrelevant study purpose (occurrence, toxicokinetic, chemistry and synthesis studies), no original data, and different meanings for BPAF. In the full text examination 39 studies were excluded. Exclusion reasons at full text examination were mainly: full text not available (conference abstracts), environmental (BPAF occurrence in soil, water) and biomonitoring (BPAF in urine, serum) studies.

**Fig. 2:**
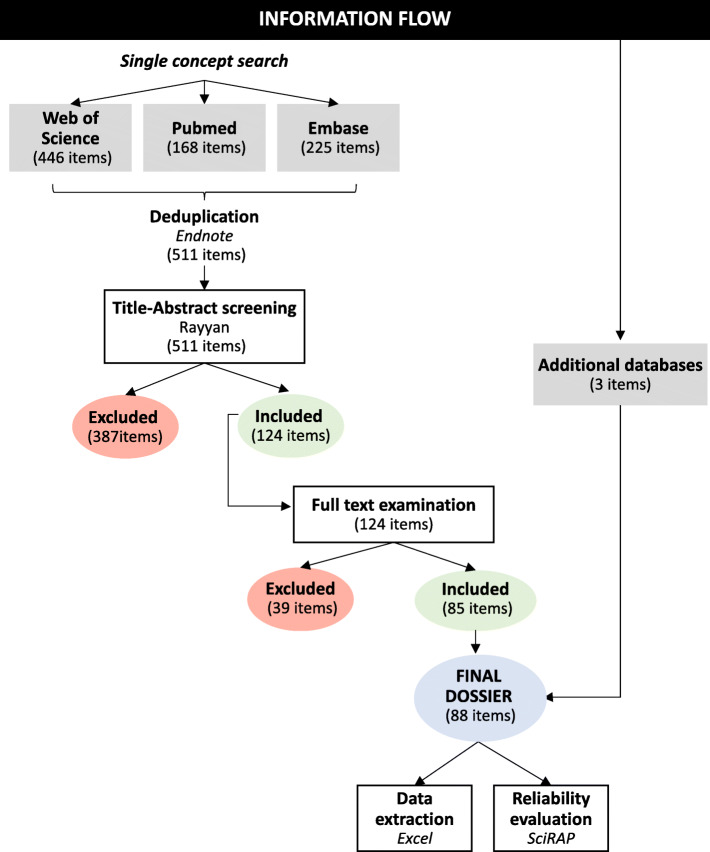
Information flow chart performed in the present study

In addition to the studies identified and selected by the search in the publication databases (85), one relevant industry study was retrieved from the REACH registration dossier, one study was identified from searches in eChemPortal, and data from the ToxCast were retrieved, obtaining 88 studies that were included in the final dossier and preliminarily classified based on the biological level of the data as follows: epidemiological (4), in silico (6), in vitro (59), in vivo/non-mammals (13), and in vivo/mammals (14). Note that some studies combine more than one type of data (eg. in vivo and in vitro), therefore the total number from the categories exceeds the number of studies (supplementary material - Appendix A). Figure 2 shows the information flow chart from the single concept search to data extraction and evaluation of studies reliability.

### Extracting and reporting the information

Data for 309 parameters were extracted from the 88 included studies and systematically reported in single rows of the excel template from the ED guidance document (supplementary material - Appendix B). The information regarding adversity included 100 parameters divided as follows: 34 EATS mediated parameters; 36 parameters sensitive to but not diagnostic of EATS; 30 parameters indicating evidence of general toxicity. With regard to endocrine activity 209 parameters were identified corresponding to 148 in vitro mechanistic in mammals, and 61 in vivo mechanistic data (34 in mammals; 27 in non-mammals).

Mammalian studies were performed in mice and rats orally exposed (but one subcutaneous) in a range of 0.05–750 mg/kg bw and exposure times between 3 and 28 days. Mammalian studies included assays such as prenatal developmental toxicity study; developmental neurotoxicity study and behaviour tests; repeated dose 28-day study; single oral dose toxicity study; adult mammalian male assay; female pubertal assay; uterotrophic assay, and Hershberger assay, among others. Three studies were based on the OECD test guidelines 407 (repeated dose 28-day oral toxicity study in rodents), and 422 (combined repeated dose toxicity study with the reproduction/developmental toxicity screening test.

In vitro assays providing mechanistic data were performed in several cell lines from mouse (mLTC-1, NIH3T3); rat (GH3); monkey (CV-1); hamster (CHO) and mainly human (BG-1FR, H295R, HeLa, HepG2, Ishikawa, MCF7, MDA-kb2, MDA-MB-231, MDA-MB-435 s, MVLN, SKBR3, T47D, U251-MG, U2OS). The in vitro assays included estrogen and androgen receptor binding and transactivation; estrogen dependent cell proliferation; estrogen dependent gene and protein expression; steroidogenesis in vitro; thyroid hormone receptor transactivation; and thyroid hormone dependent gene expression. The exposure doses and times ranged between 0.000001–1000 μM and 1 h-2 days, respectively. One study was based on OECD guideline 455 (stably transfected transactivation in vitro assays to detect estrogen receptor agonists and antagonists). Additional mechanistic data were obtained from ToxCast studies, in silico prediction models, yeast bioassays and proteins expressed using virus and *E. coli*.

Non-mammalian mechanistic data included studies in fish (medaka and mainly zebrafish) measuring vitellogenin and other estrogen dependent gene and protein expression, steroidogenesis gene expression, estradiol and testosterone levels, thyroid hormone dependent gene expression and thyroid hormone levels. Exposure times ranged between 8 h and 120 days and concentrations ranged between 0.001–17 mg/L water.

### Evaluating reliability

Reliability evaluation allowed classification of studies into three categories: reliable, partially reliable and not reliable. The studies containing in vitro data were rated as reliable (72%), partially reliable (19%), and not reliable (9%). The studies containing in vivo assays performed in mammalian species were assessed as reliable and partially reliable (86 and 14%, respectively) while those performed in non-mammalian species were rated as reliable (69%), partially reliable (23%), and not reliable (8%). Note that some studies were assessed twice for both in vitro and in vivo*.* The studies’ ID, title, publication link and reliability results are shown in supplementary material - Appendix A.

### Assembling lines of evidence

**Fig. 3:**
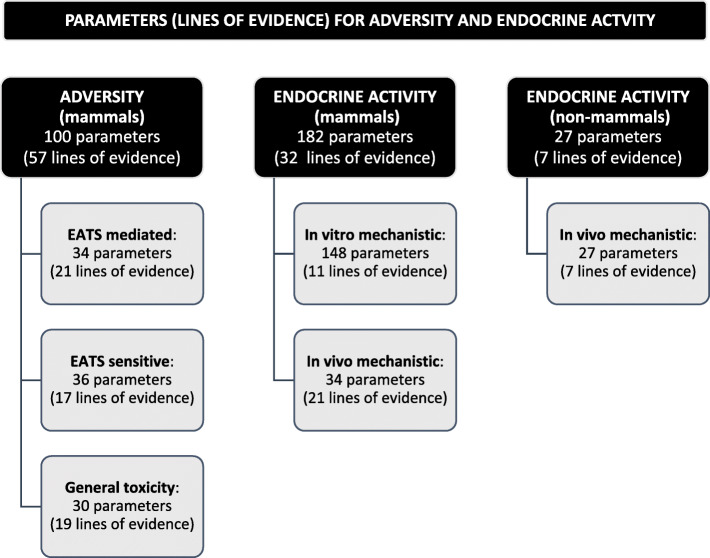
Number of extracted parameters and assembled lines of evidence for adversity and endocrine activity

The 309 extracted parameters were assembled into 96 lines of evidence that were organized in groups and subgroups as explained in section 2.6 (supplementary material – Appendix B). As it is shown in Fig. 3 the data supporting evaluation of adversity included 57 lines of evidence in mammals, and data supporting evaluation of endocrine activity included 39 lines of evidence (11 in vitro mechanistic in mammals, 21 in vivo mechanistic in mammals; and 7 in vivo mechanistic in non-mammals). Parameters indicating evidence of general toxicity were assembled in 19 lines of evidence.

### Assessing, integrating and reporting lines of evidence

Lines of evidence for adversity and endocrine activity were assessed separately for mammals and non-mammals. An overview of the WoE evaluation of lines of evidence for adversity and endocrine activity evaluated in mammals is illustrated in Tables 3 and 4, respectively. Complete information about lines of evidence for endocrine activity and adversity is shown in supplementary material – Appendix B.

**Table 3:** Overview of the integrated lines of evidence for adversity in mammals, classified by the weight of evidence evaluation as strong, moderate, weak, or no evidence for effect and the study IDs. For detailed information and data on general toxicity, see supplemental material - Appendix B

Lines of evidence for ADVERSITY in mammals		
Weight of Evidence	EATS mediated parameters	Parameters sensitive to but not diagnostic of EATS
*Strong*	■Estrous cycling disruption (adult exposure) IDs: 60, 83, 88 ■Mammary gland histopathology alteration in female (developmental exposure) ID: 59 ■Ovary histopathology alteration (adult exposure) IDs: 83, 88 ■Testis histopathology alteration (adult exposure) IDs: 60, 88 ■Prostate weight decrease (adult exposure) ID: 60 ■Epididymis weight decrease (adult exposure) ID: 88 ■Seminal vesicles weight decrease (adult exposure) ID: 60 ■Seminal vesicles histopathology alteration (adult exposure) ID: 88	■Brain weight increase in male and female (adult exposure) IDs: 60, 88 ■Adrenals weight increase in male (adult exposure) IDs: 60, 88 ■Adrenals histopathology alteration in male (adult exposure) IDs: 60, 88 ■Pituitary gland histopathology alteration in male (adult exposure) IDs: 60, 88 ■Fertility decrease in male (adult exposure) ID: 88 ■Developmental neurotoxicity: behaviour alterations ID: 57
*Moderate*	■Mammary gland histopathology alteration in female (adult exposure) IDs: 60, 88 ■Mammary gland histopathology alteration in male (adult exposure) IDs: 60, 88 ■Ovary weight decrease (adult exposure) IDs: 83, 88	
*Weak*	■Testis weight decrease (adult exposure) IDs: 55, 88	■Adrenals histopathology alteration in female (adult exposure) IDs: 60, 88
*No evidence for effect*	■Estrous cycling alteration (developmental exposure) ID: 59 ■Uterus weight alteration (pregnant exposure) ID: 88 ■Uterus histopathology alteration (pregnant exposure) ID: 88 ■Vaginal opening alteration (gestational exposure) ID: 59 ■Vagina histopathology alteration (adult exposure) ID: 88 ■Testis weight alteration (developmental exposure) ID: 58 ■Epididymis weight alteration (developmental exposure) IDs: 58, 84 ■Sperm morphology alteration (adult exposure) ID: 60 ■Thyroid weight alteration in male and female (adult exposure) IDs: 60, 88	■Adrenals weight alteration in females (adult exposure) IDs: 60, 88 ■Pituitary gland histopathology alteration in females (adult exposure) IDs: 60, 88 ■Time to mating alteration in males and females (adult exposure) ID: 88 ■Gestation length alteration (gestational exposure) ID: 88 ■Sex ratio alteration (gestational exposure) ID: 58 ■Litter size alteration (gestational exposure) IDs: 56, 58, 84, 88 ■Litter viability alteration (gestational exposure) IDs: 56, 58 ■Litter/pup weight alteration (gestational exposure) ID: 58 ■Pup survival index alteration (gestational exposure) ID: 58 ■Pup development alteration (gestational exposure) ID: 88

**Table 4:** Overview of the integrated lines of evidence for endocrine activity in mammals, classified by the weight of evidence evaluation as strong, moderate, weak, or no evidence for effect and the study IDs. For detailed information and data on general toxicity, see supplemental material - Appendix B

Lines of evidence ENDOCRINE ACTIVITY in mammals		
Weight of Evidence	In vitro mechanistic	In vivo mechanistic
*Strong*	■Estrogen receptor binding-in silico IDs: 45, 51, 72, 75 ■Estrogen receptor binding and agonist activity IDs: 3, 6, 8, 11–15, 17–18, 20–22, 42–44, 46, 48, 52, 62–65, 78, 81, 86–87 ■Estrogen dependent cellular proliferation IDs: 7–6, 10, 15, 20, 48, 80 ■Estrogen receptor dependent gene/protein expression increased IDs: 9–13, 15, 17–18, 20, 23, 48, 80 ■Androgen receptor antagonist binding-in silico IDs: 75–76 ■Androgen receptor binding and antagonist activity IDs: 19, 21–22, 43, 64, 78, 81, 87 ■Steroidogenesis alteration IDs: 4–5, 87 ■Thyroid hormone receptor binding-in silico ID: 75 ■Thyroid hormone related gene expression decreased ID: 7	■Estrogen receptor dependent gene expression increased (adult and gestational exposure) IDs: 59, 83 ■Uterus weight increase (adult exposure) IDs: 61–63, 86 ■Uterus histopathology alteration (adult exposure) ID: 63 ■Steroidogenesis gene/protein expression alteration (adult and gestational exposure) IDs: 55, 58, 83 ■Estradiol level increase in female offspring (gestational exposure) ID: 59 ■Testosterone level decrease in male (adult exposure) ID: 55 ■Progesterone level increase in female offspring (gestational exposure) ID: 59 ■Progesterone level decrease in female (adult exposure) ID: 83 ■FSH level increase in male (adult exposure) ID: 55 ■LH level increase in male (adult exposure) ID: 55 ■T4 level increase (adult exposure) ID: 60
*Moderate*	■Thyroid hormone receptor activity alteration IDs: 7–8, 21, 64	■Testosterone level increase in male offspring (gestational exposure) ID: 58
*Weak*		■Weight increase of male androgen-dependent sex accessory tissues (adult exposure ID: 61 ■Testosterone level decrease in female offspring (gestational exposure) ID: 59
*No evidence for effect*		■Estradiol level alteration in male offspring (gestational exposure) ID: 58 ■Estradiol level in female (adult exposure) ID: 83 ■Testosterone fetal production alteration (gestational exposure) ID: 56 ■FSH level alteration in female (adult exposure) ID: 83 ■FSH levels alteration in male offspring (gestational exposure) ID: 58 ■LH level alteration in male offspring (gestational exposure) ID: 58 ■TSH level alteration (adult exposure) ID: 60

Lines of evidence for adversity were grouped into: i) EATS-mediated parameters; ii) parameters sensitive but not diagnostic of EATS; and iii) evidence for general toxicity (supplementary material – Appendix B).

EATS-mediated parameters included estrous cycling alteration that was reported in adult rats (30 mg/kg bw; 28 days; ID 60) showing estrous cycle irregularities and (not statistically significant) diestrous stage prolongation; and in mice (90 mg/kg bw; 6 weeks; ID 83) that showed estrous cycle detention with prolonged metestrus/estrus stage. Alterations in mammary gland histopathology were observed in female mice after fetal exposure (0.5–5 mg/kg/day; 8 days; ID 59) at all doses showing accelerated pubertal mammary gland development and late mammary gland lesions observed in offspring. Ovaries histopathology alteration was observed in adult mice (90 mg/kg bw; 6 weeks; ID 83) showing an increase of dead atretic follicles and decrease of secondary follicles and corpora lutea number; as well as ovary cysts observed in adult rats (300 mg/kg bw; 55 days; ID 88). Testis histopathology alteration was reported in rats after 28 days (100 mg/kg bw; ID 60) and 55 days (300 mg/kg bw; ID 88) of adult exposure both showing atrophy of testicular Leydig cells. A decrease in absolute and relative prostate weight (100 mg/kg bw; 28 days; ID 60) and absolute and relative epididymis weight (300 mg/kg bw; 55 days; ID 88) was observed in adult rats after oral exposure. Seminal vesicles absolute weight was decreased in rats after oral exposure (100 mg/kg bw; 28 days; ID 60), while an alteration of seminal vesicles histopathology was shown also in rats (300 mg/kg bw; 55 days; ID 88) with reduced secretory content as indicated by smaller organ size.

Other lines of evidence for EATS-mediated parameters assessed as moderate WoE in mammals included: changes in mammary gland histopathology (female, adult exposure), mammary gland histopathology (male, adult exposure), and ovary weight (adult exposure); while data for decreased testis weight (adult exposure) showed weak WoE.

Parameters sensitive but not diagnostic of EATS included increase in brain relative weight (male and female) and adrenals relative weight (male) that were observed in rats after 28 days (100 mg/kg bw; ID 60) and 55 days (30–300 mg/kg bw; ID 88) oral exposure. Adrenals histopathology alteration was observed in male rats with adrenal gland hypertrophy of zona fasciculata (100 mg/kg bw; ID 60) and cortical vacuolation significantly less prevalent (300 mg/kg bw; ID 88). Pituitary gland histopathology alteration was observed in male rats with reduced vacuolation of pars anterior cells (300 mg/kg bw; 55 days; ID 88) and atrophy of basophilic cells (100 mg/kg bw; ID 60). Reduced fertility (30 mg/kg bw) and infertility (100 mg/kg bw) was reported in male rats after 55 days oral exposure (ID 88). Developmental neurotoxicity effects were observed in mice by several tests after oral exposure (0.4 mg/kg bw; 9 days; ID 57) including decrease of time spent in central zone (males); feeding latency increase (anxiety-like behaviors) in males and decrease (anxiolytic effects) in females; decreased sucrose preference (males); increased immobility time (males); impaired novel objects recognition memory formation after long-term (24 h) in males and females but no effect after short term (1.5 h); and impaired contextual fear memory formation after short and long-term (1.5 h and 24 h) in male. However, no effect was observed in total food intake, floating time by forced swimming test, recognition index by short term memory test, freezing time by short-term and long-term memory test. Changes in adrenals histopathology (females) was assessed as weak WoE.

Supportive information encompassed epidemiological studies that were assessed separately as supportive information (data not shown). These studies reported significant correlation with risk of differentiated thyroid cancer and strong relationship with malignancy in 55 patients; 27 benign thyroid nodules and 28 differentiated thyroid cancer (study ID 73); positive association with type II diabetes mellitus in 251 patients vs. 251 controls (study ID 70); significant higher serum concentration rates in donors with abnormal fasting blood glucose levels found in e-waste recycling areas (*n* = 119 patients) than those found in a reference area (*n* = 16 donors) (study ID 74). One study investigated pregnancy exposure and relations to steroid changes but did not observe signs of transplacental transport in 27 pregnant women (ID 71).

Lines of evidence for general toxicity in mammals evaluated as strong evidence included increase of absolute but not relative heart weight in females, increase of relative thymus weight in males, kidney histopathology alteration (basophillic tubules and tubular dilatation) in males, and gross morphology alteration (large intestinal lumen dilatation) in males and females. Effects of general toxicity assessed as weak evidence included heart, kidney, liver and spleen weight alterations in males, liver histopathology alteration in females and total cholesterol as well as moderate evidence of liver histopathology alteration in males. No evidence for effect was reported for kidney, liver, spleen and thymus weight (females), liver weight (developmental exposure), kidney histopathology (females), spleen and bone marrow histopathology (males and females) (supplementary material – Appendix B).

Lines of evidence for endocrine activity in mammals were grouped into: i) in vitro mechanistic; and ii) in vivo mechanistic data (supplementary material - Appendix B).

In vitro mechanistic data included estrogen receptor binding that was shown in silico (IDs 45, 51, 72, 75) and using receptor protein (IDs 14, 20, 62, 65, 86). Estrogen receptor agonistic activity via ERα or ERβ was shown in hamster (CHO-K1; ID 78), monkey (CV-1; ID 22), and human cells including HeLa, HepG2, MCF-7, BG-1FR, Ishikawa/ERα, T47D-Kbluc, MVLN, U251-MG, MDA-MB-231, SK-BR-3 (IDs 3, 11, 12, 13, 14, 15, 17, 21, 46, 52, 62, 63, 65), as well as in yeast assays (IDs 6, 8, 42, 43, 81). ToxCast ER prediction model was also positive (IDs 87). In some studies, no activation of ERβ was observed (IDs 11, 12, 17). Estrogen receptor antagonistic activity was reported in some studies (IDs 11, 14, 18, 78), whereas others were negative (IDs 11, 21, 22, 62), possible due to cell or receptor specificity.

Estrogen dependent cell proliferation was increased in rat cells (GH3; ID 7) and human hormone dependent cells (MCF-7, T47D; IDs 6, 10, 15, 20, 48, 80) showing in some cases potentiated effect with estradiol co-exposure, reversed effect with ERα inhibitors, and cell proliferation decrease after blocking.

ER receptors. Moreover, no cell proliferation was shown in ERα negative cells (MDA-MB-231; ID 15).

Estrogen receptor dependent gene and protein expression was shown in several cell lines. Induction of estrogen receptor dependent gene expression, some in dose and time dependent manner, was shown in human cells (T47D, MCF-7, Ishikawa/ERα, HeLa, MDA-MB-231; ID 9, 10, 11, 12, 13, 15, 17, 18, 23, 48) including estrogen related genes such as CXCL12, TFF1, TFF2, CTSD, PGR, pS2, GREB, GREB1, ERβ, ERβ1, ERβ2, Egr-1, SPUVE, WISP2, SDF-1, WISP-2/CNN5, RIP140, MYB, MGP, MYB-AS1, AGR3. Moreover, reversed effects were shown when co-exposure with an ER antagonist (ICI) (ID 13). With regard to protein expression, induction of PGR, ERα, ERβ, GPER, pS2, Cyclin D1, and c-Myc protein was shown in human cells (MCF-7; ID 18, 20, 80) with effects reduction by ERα inhibitor ICI.

Androgen receptor binding was shown in silico (ID 75, 76). Similar AR binding profile as an AR antagonist (CPA), but different than the agonist synthetic androgen (R1881), was shown in human cells (U2OS; ID 19) indicating anti-androgenic (AR antagonistic) but not androgenic (AR agonistic) activity. Anti-androgenic (and no androgenic activity) was shown in yeast (IDs 43, 81), mouse (NIH3T3; ID 64), hamster (CHO-K1; ID 78), monkey (CV-1; ID 22), and human (MDA-KB2; ID 21) cells in some cases in a dose dependent manner, also showing competitive antagonism with a synthetic AR agonist (R1881). ToxCast prediction models were positive for AR antagonist and negative for AR agonist (ID 87).

Effects in steroidogenesis were observed by positive ToxCast steroidogenesis assays (NVS_ADME_hCYP19A1 and TOX21_Aromatase_Inhibition; ID 87), as well as in vitro by the alteration of steroidogenic hormone levels such as dose dependent decrease of testosterone, aldosterone and cortisol; and progesterone level increase in human (H295R) cells (ID 4). Dose dependent decrease of progesterone level was observed instead in mouse (mLTC-1) cells (ID 5) and both human and mouse cells showed alteration of steroidogenic gene and protein expression including dose dependent suppression of the genes CYP17A1, CYP11B2, HSD3B2, CYP11B1, FDX-1, P450scc, SR-B1, StAR and expression decrease of the steroidogenic proteins SR-B, P450scc (ID 4 and 5).

Thyroid hormone receptor binding for agonist effect was observed in silico (ID 75), and a decrease of thyroid hormone related gene (Tshβ, Trα, Trβ, Dio1, Dio2) expression was observed in rat cells (GH3; IDs 7), while thyroid hormone receptor activity was assessed as moderate WoE (ID 7, 8, 21, 87).

In vivo mechanistic data included alteration of ER dependent gene expression that was observed in adult mice after oral exposure (3–90 mg/kg bw; 6 weeks; ID 83) with uterus PGR gene expression increase at all doses, as well as in offspring mice after gestational exposure (0.05–5 mg/kg bw; 8 days; ID 59) showing increase in mammary gland gene expression of Esr1, PGR.

Dose dependent increase of absolute and relative uterus weight was observed in rats after subcutaneous exposure (8 mg/kg bw; 3–4 days; IDs 61, 62, 86), while uterus weight increase and histopathology alterations (cell height increase and columnar differentiation) were observed in rats after oral exposure (50 mg/kg bw; 4 days; ID 63) in uterotrophic assays.

Alteration of steroidogenic gene expression was observed in adult mice after oral exposure (3–90 mg/kg bw; 6 weeks; ID 83) with decrease of uterus gene expression (Cyp11a1, StAR), as well as in adult rats after oral exposure (200 mg/kg bw; 14 days; ID 55) showing a decrease in testis gene (SR-B1, StAR, P450scc, 17β-HSD, ER-α, LHR, Inhibin B, SREBP-1c) and protein (SR-B1, StAR, P450scc) expression. Testis gene expression decrease (ERα, AR, StAR) and protein expression alteration (P450scc, StAR, AR, PTPRJ, DPYSL3) were observed in rats after 17 days gestational plus 17 days post-natal exposure (100 mg/kg bw; ID 58).

Several hormone levels were altered in mammals including estradiol and progesterone increase in female mice after gestational exposure (0.05 mg/kg bw; 8 days; ID 59); progesterone decrease in female adult mice (90 mg/kg bw; 6 weeks; ID 83); testosterone decrease, FSH and LH increase in adult male rats (2–200 mg/kg bw; 14 days; ID 55); and T4 level increase in male and female adult rats (100 mg/kg bw; 28 days; ID 60). Testosterone level increase in male offspring rats was assessed as moderate WoE, while lines of evidence reported as weak WoE included: weight of male androgen-dependent sex accessory tissues and testosterone level in female offspring-gestational exposure (mammalian).

### Initial analysis of the evidence

Considering the available information in the dataset, EATS-mediated adversity in mammals was observed for EAS modalities although it was not sufficiently investigated (ED guidance document scenario 1b). EATS-mediated endocrine activity was observed for EAS but it was not conclusive for T modality. EATS-mediated endocrine activity was sufficiently investigated for E modality (ToxCast ER bioactivity model - ID 87; and uterotrophic assay - IDs 61, 62, 63, 86), and for A modality (Herschberger assay - ID 61). Endocrine activity was not sufficiently investigated for T modality (only OECD TG 407; ID 60) and for S modality (H295R steroidogenesis assay TG456 - ID 4; but absence of aromatase assay OPPTS890.1200). Effects supporting conclusions about adversity and endocrine activity took place in the absence of general toxicity. The biological plausibility of the link between the EATS-mediated adversity and endocrine activity should be documented through MoA postulation according to the ED guidance document.

### Mode of action postulation

**Fig. 4:**
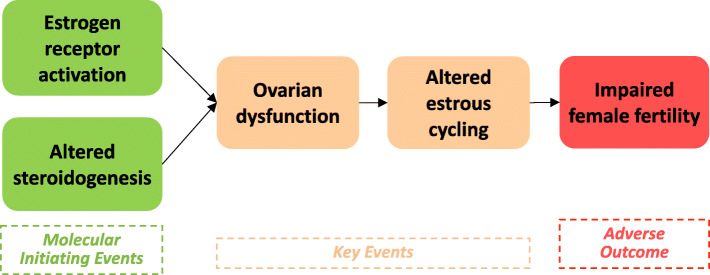
Postulated MoA for BPAF in female adult exposure

**Fig. 5:**
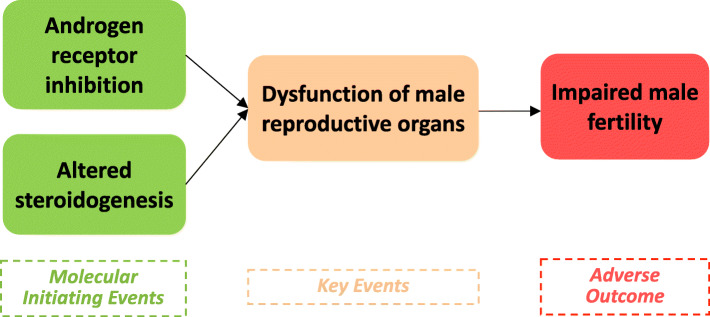
Postulated MoA for BPAF in male adult exposure

Although a single MoA postulation is sufficient according to the ED guidance document, in the present study two MoAs for BPAF were postulated for effects of adult exposure on female and male reproduction, respectively (Figs. 4 and 5). These MoAs were built based on lines of evidence assessed as strong and moderate WoE from the present dataset.

The MoA for effects on female reproduction is postulated to be initiated by two MIE, estrogen receptor activation and altered steroidogenesis. Two parallel MIEs are suggested, since current knowledge in endocrinology does not provide sufficient knowledge to conclude whether they are indeed parallel or whether one of them precedes the other. The first MIE, estrogen receptor activation, is supported by in vitro mechanistic data on estrogen receptor binding, estrogen receptor agonist activity and increased estrogen dependent cellular proliferation and estrogen receptor dependent gene/protein expression. The MIE is also supported by in vivo mechanistic data on increased estrogen receptor dependent gene expression, uterus weigh increase and uterus histopathology alteration. The second MIE, altered steroidogenesis, is supported by in vitro mechanistic data on effects on steroidogenesis, and in vivo mechanistic data on alteration of steroidogenesis gene and protein expression and decreased progesterone levels. The two MIEs are postulated to lead to KE ovarian dysfunction. The KE is supported by the EATS-mediated parameters ovary histopathology alteration and ovary weight decrease. The following KE is altered estrous cycling that is supported by the EATS-mediated parameter altered disruption of estrous cycling. The postulated AO is impaired female fertility. No BPAF data to support the AO were identified but it is hypothesised based on current knowledge in endocrinology. The postulated MoA for BPAF in female adult exposure is shown in Fig. 4. Brief description and supporting evidence of the included KEs are shown in Table 5.

**Table 5:** Description and supporting evidence of the KEs included in postulated MoA for BPAF in female adult exposure

	Brief description of key event	Supporting evidence
**MIE 1**	Estrogen receptor activation (two parallel MIEs are suggested, current knowledge in endocrinology does not provide sufficient knowledge to conclude whether they are indeed parallel or whether one of them precedes the other)	In vitro mechanistic: ■Estrogen receptor binding IDs: 45, 51, 72, 75 ●Estrogen receptor agonist activity IDs: 3, 6, 8, 11–15, 17–18, 20–22, 42–44, 46, 48, 52, 62–65, 78, 81, 86–87 ●Estrogen dependent cellular proliferation IDs: 7–6, 10, 15, 20, 48, 80 ■Estrogen receptor dependent gene/protein expression increased IDs: 9–13, 15, 17–18, 20, 23, 48, 80 In vivo mechanistic: ■Estrogen receptor dependent gene expression increased (adult exposure) IDs: 59, 83 ●Uterus weight increase (adult exposure) IDs: 61–63, 86 ●Uterus histopathology alteration (adult exposure) ID: 63
**MIE 2**	Altered steroidogenesis	In vitro mechanistic: ●Steroidogenesis alteration IDs: 4–5, 87 In vivo mechanistic: ●Steroidogenesis gene/protein expression alteration (adult exposure) IDs: 55, 58, 83 ●Progesterone level decrease in female (adult exposure) ID: 83
**KE1**	Ovarian dysfunction	EATS-mediated: ●Ovary histopathology alteration (adult exposure) IDs: 83, 88 ●Ovary weight decrease (adult exposure) IDs: 83, 88
**KE2**	Altered estrous cycling	EATS-mediated: ●Estrous cycling disruption (adult exposure) IDs: 60, 83, 88
**AO**	Impaired female fertility	None (no data available, but hypothesized based on current knowledge in endocrinology)

The MoA for effects on male reproduction is also postulated to be initiated by two MIE, androgen receptor inhibition and altered steroidogenesis. Two parallel MIEs are suggested, on the same basis as for the MIEs in the female MOA. The first MIE, androgen receptor inhibition, is supported by in vitro mechanistic data on androgen receptor antagonist binding and antagonist activity. The MIE is also supported by in vivo mechanistic data on decreased weight of prostate, epididymis and seminal vesicles. The second MIE, altered steroidogenesis, is supported by in vitro mechanistic data on effects on steroidogenesis, and in vivo mechanistic data on alteration of steroidogenesis gene and protein expression and decreased testosterone levels and increased FSH and LH levels. The two MIEs are postulated to lead to the KE dysfunction of male reproductive organs. The KE is supported by the EATS-mediated parameters histopathology alteration of testis and seminal vesicles, as well as decrease of prostate, epididymis and seminal vesicles weight. The postulated AO is impaired male fertility. The AO is supported by the sensitive but not diagnostic of EATS parameter decreased fertility. The postulated MoA for BPAF in male adult exposure is shown in Fig. 5. Brief description and supporting evidence of the included KEs are shown in Table 6.

**Table 6:** Description and supporting evidence of the KEs included in postulated MoA for BPAF in male adult exposure

	Brief description of key event	Supporting evidence
**MIE 1**	Androgen receptor inhibition (two parallel MIEs are suggested, current knowledge in endocrinology does not provide sufficient knowledge to conclude whether they are indeed parallel or whether one of them precedes the other)	In vitro mechanistic: ●Androgen receptor antagonist binding IDs: 75–76 ●Androgen receptor antagonist activity IDs: 19, 21–22, 43, 64, 78, 81, 87 In vivo mechanistic: ●Prostate weight decrease (adult exposure) ID: 60 ●Epididymis weight decrease (adult exposure) ID: 88 ●Seminal vesicles weight decrease (adult exposure) ID: 60
**MIE 2**	Altered steroidogenesis	In vitro mechanistic: ●Steroidogenesis alteration IDs: 4–5, 87 In vivo mechanistic: ●Steroidogenesis gene/protein expression alteration (adult exposure) IDs: 55, 58, 83 ●Testosterone level decrease in male (adult exposure) ID: 55 ●FSH level increase in male (adult exposure) ID: 55 ●LH level increase in male (adult exposure) ID: 55
**KE1**	Dysfunction of male reproductive organs	EATS mediated: ●Testis histopathology alteration (adult exposure) IDs: 60, 88 ●Prostate weight decrease (adult exposure) ID: 60 ●Epididymis weight decrease (adult exposure) ID: 88 ●Seminal vesicles weight decrease (adult exposure) ID: 60 ●Seminal vesicles histopathology alteration (adult exposure) ID: 88
**AO**	Impaired male fertility	Sensitive but not diagnostic of EATS: ●Fertility decrease (adult exposure) ID: 88

### Overall conclusion on the ED criteria

Based on this assessment, it is concluded that BPAF shows EAS-mediated endocrine activity and EAS-mediated adversity. A biologically plausible link between endocrine activity and adversity was established using MoA analysis for both impaired male and impaired female fertility. Thus, BPAF meets the ED criteria for EAS modalities.

## Discussion

While scientific criteria and guidance for the identification of EDs are now in place for PPPs and BPs in the EU, no such specific process for identifying ED properties of chemicals regulated under other legislations, such as REACH, is yet established. The EU Commission has communicated that a horizontal approach harmonizing ED assessment across different EU legislation should developed, building on the ED criteria implemented for PPPs and BPs [3]. In response, the EU Parliament emphasised the need for a swift harmonization and implementation of an EU approach for EDs, which should include definitions for known and presumed EDs in line with the classification of carcinogenic, mutagenic and reprotoxic substances (CMRs) in the EU Regulation for Classification, Labelling and Packaging [26]. It is therefore highly relevant to investigate the applicability of the ED criteria and corresponding guidance to the regulation of non-pesticide compounds. As recently reported the ECHA/EFSA guidance can and should be applied to all chemicals independent of their intended application [27]. In this study, the process for assessment of ED properties established for PPPs and BPs in the EU was applied in a case study using BPAF as a model substance.

Although some challenges and limitations were faced and identified during the assessment, all the steps described in the ED guidance document were successfully completed, resulting in a thorough, structured and transparent identification of BPAF as an ED. The initial analysis of the data evaluated in the present study indicated that BPAF exposure may result in different types of EAS-mediated adversity in mammals, and that it shows endocrine activity for the EAS-modalities, primarily. The biological plausibility of the link between the adversity and endocrine activity was subsequently documented by MoA postulation, which supported that adult exposure to BPAF may induce female and male impaired fertility by interfering with estrogen and androgen signalling, respectively. Overall, the conclusion from the ED assessment was that BPAF meets the ED criteria, showing disruption in EAS modalities. In line with these results, a previous report from the Technical University of Denmark concluded that BPAF was a suspected ED substance, based on the WHO definition of an ED [28]. The DTU report judged that there was strong evidence of E-mediated endocrine adverse effects (delay in male puberty, advancement in female puberty and clear effects on fertility) and of an endocrine MoA, based on in vitro and in vivo data, as well as a strong plausible link between the MoA and the adverse effects.

The present work illustrates the application of the principles for ED assessment of pesticides described in the ED guidance document in the assessment of a REACH chemical. A supposition of this work was that since regulatory requirements for the testing and assessment of chemicals differ between these regulatory frameworks, the process for ED assessment may need to be adjusted to fit the requirements under REACH. The regulatory information requirements for pesticides include a number of standardized studies that are useful for the EDs assessment. Requirements for PPPs are the most extensive, including repeated dose toxicity tests in two species, chronic exposure, developmental toxicity in two species and reproductive toxicity test [29]. Information requirements for BPs include repeated dose toxicity tests (28 day and/or 90 day), chronic exposure and reproductive toxicity tests; pre-natal developmental toxicity studies, and/or a two-generation reproductive toxicity test [30]. Still, it is foreseen that this information may not always be sufficient to complete the assessment of ED potential and the lack of in vitro and in vivo mechanistic data may be specifically critical since apical findings from in vivo data often do not enable a mechanistic understanding of the observed AO [31]. Mechanistic information is only available from a few required studies for pesticides and may especially be insufficient if the study was conducted according to older test guidelines. Mechanistic data are essential in the ED assessment process, as it provides the basis for evaluating endocrine activity and for MoA analysis. The ED criteria stipulate that all information relevant for ED assessment should also be collected from other sources [9, 10], such as databases and the open scientific literature, and this may be especially important in order to gain sufficient mechanistic data. However, it must be acknowledged that such data may often not be available, especially for new substances.

Testing requirements under REACH [12] are in general less extensive than for pesticides and are set according to the amount of chemical (tonnes per year) that is either produced in or imported to the EU. Annexes VII to X of the REACH Regulation contain the standard information requirements for the different tonnage bands, from 1 to 10 t/year to > 1000 t/year. For many, in particular low tonnage substances, non or few complex toxicity studies are required. For example, repeated dose toxicity tests (28 day) and screening tests for reproductive/developmental toxicity are required for chemicals produced or imported at or above 10 t per year with more complex tests, such as reproductive toxicity tests, required for chemicals produced or imported at or above 100 and 1000 t/year. Data for investigating toxicological mechanisms other than mutagenicity are not required as regard low tonnage bands. This raises concern that the toxicological information available for the majority of REACH chemicals will rarely result in sufficient information to perform the ED assessment.

There is currently a rapid global development of non-animal (e.g. in silico, *in chemico* and in vitro) methods for testing chemicals, driven by stakeholder needs, academic research interests and increased regulatory focus on the 3R (refine, reduce and replace animal testing) concept [32–34]. Currently, regulatory use of such novel methods is often hampered by a lack of test validation, as well as a limited understanding of the mechanistic connections between what is tested and any adverse outcomes. Nevertheless, such methods have the potential to provide critical mechanistic information in a resource-efficient manner, which is especially essential for the assessment of ED. For example, concerning receptor interactions, enzyme activation or inhibition affecting hormone synthesis, or interference with the function of transport proteins. Recent guidance from the OECD provide support for the development and execution of new in vitro methods to ensure regulatory applicability [32–34].

Given the issues of insufficient regulatory information requirements, availability of data from other sources, e.g. the open literature and databases such as ToxCast, becomes crucial for ED assessment, especially for chemicals regulated only under REACH. It is acknowledged that BPAF is a relatively data-rich substance, indeed the availability of data was one of the considerations for choosing it for this case study. The standard test data from OECD studies for this substance include several repeated-dose toxicity tests and multigeneration reproductive toxicity tests, as well as both the Hershberger and uterotrophic assays. In addition, a relatively large amount of relevant data was available from the open literature. The case presented in this study may therefore not be representative of the majority of REACH chemicals. Realistic scenarios where available scientific information is limited should be considered in future case studies to further explore the regulatory consequences when data are insufficient for evaluating endocrine activity or adversity, or to conduct MoA analysis. However, this case study raises the important point of how to collect, consider and evaluate the relevance and reliability of mechanistic and toxicological data that were not generated in accordance with standardised test guidelines. There are several aspects related to the guidance for WoE evaluation in the current ED guidance document that can be further developed and improved in order to give more concrete guidance for its intended users.

The ED criteria for PPPs and BPs state that all “other” data relevant for the ED assessment, i.e. data available from the open scientific literature and from databases, should be selected using systematic review methodology. In addition, all available evidence, including both data from the regulatory testing and from “other” sources, should then be assessed based on a WoE approach. Recently published guidance from EFSA [24] and from the European Commission Scientific Committee on Health, Environmental and Emerging Risks [25] are available and describe general principles for WoE evaluation in the context of health risk assessment. The main steps of WoE evaluation can be summarized as collecting the evidence, evaluating individual studies and lines of evidence, and integration of the different lines of evidence. The ED guidance document gives an overall description of how to gather, evaluate and consider all relevant information for the assessment. However, a specific WoE evaluation approach is not described. The ED guidance document gives an overview on the information sources and how to consider the scientific data generated for ED identification. However, the guidance for WoE evaluation is mainly focused on the standardized test guidelines described in OECDs guidance and framework for evaluating chemicals for ED No. 150 [11]. This is understandable since the assessment of pesticides is mainly reliant on the regulatory testing conducted according to standardized guidelines. However, as discussed above, the inclusion of any available data from the open literature, which are often non-standard data, is essential for ED assessment given the limitations of the current regulatory information requirements. Especially for REACH chemicals. Indeed, in some cases like in the present case study, non-standard data could represent the great majority of the available data.

Systematic review methodology is primarily mentioned in the context of ED assessment in the step for collecting relevant evidence. From the scientific perspective, the application of systematic review methodology provides several advantages in terms of ensuring that all relevant evidence is being considered in a structured and transparent manner. However, for conducting ED assessment in the regulatory setting, application of systematic review methodology may be challenging, primarily because of the amount of time required. The development of systematic methods for application in environmental health, e.g. to answer questions regarding the connection between exposure to environmental factors and adverse health effects, is receiving increasing interest from the research community [35–38], as well as regulatory agencies and expert organs [22, 39–41]. Systematic review methodologies have mainly been developed and applied in the field of medicine and their use in regulatory risk assessment of chemicals is still relatively new. The benefits of applying systematic review include increasing transparency and structure in how evidence is collected and evaluated to answer questions about health effects from exposure to environmental chemicals. This improves the robustness of the scientific basis for regulatory decisions, such as allowing or restricting the use of certain chemicals. However, systematic review methodology has to be adjusted to be fit for purpose for the evaluation of health effects of environmental factors. Challenges in this field include how to systematically integrate different types of evidence, i.e. data from both epidemiological and toxicological (in vivo*,* in vitro*,* in silico) studies to reach an overall conclusion regarding, for example, the health hazards of a specific environmental chemical [35, 36].

In order to gather all information relevant for ED assessment, a systematic and exhaustive search of the open literature and relevant databases should be performed. The primary approach is to use a single concept strategy [7]. However, this approach may in some cases result in too many (irrelevant) hits, requiring further refinement of the search by running a targeted search on specific terms. Designing a proper targeted search strategy, which enables capturing all relevant information, is essential to avoid bias in the ED assessment. The ED guidance document Appendix F includes recommendations for a targeted search strategy. However, while conducting this case study needs to improve the targeted search were identified. Refined search filters that balance high sensitivity and high specificity were developed and validated and have been recently published [42]. According to systematic review methodology, the screening of data to decide on inclusion or exclusion in the assessment should be conducted by two separate reviewers and when conflicts between the reviewers arise these should be resolved in a transparent manner [22, 43]. This process was followed in the current case study, although not specifically required or described in the ED guidance document. In the regulatory setting, where time and resources may be limited it is likely that decisions on inclusion/exclusion of data will be based on the decision of one reviewer. It should be noted that this is considered a serious limitation from the perspective of systematic review methodology.

In the ED guidance document little guidance is provided on how to evaluate the relevance and reliability of data generated in studies not conducted according to standardized test guidelines. In this case study, the SciRAP tool [23, 44] was used to perform structured and consistent evaluation of the reliability of the collected data. Although this method for data evaluation is time consuming, a structured approach is critical to ensure systematic and transparent evaluation of the available evidence. The Klimisch approach [45] is commonly used in the regulatory setting in Europe to categorize data into different reliability categories. However, this approach does not provide transparent criteria for categorization. The SciRAP tool can be used for categorizing studies into Klimisch cateogories if needed, as shown previously [46].

In addition, even if no such instructions are explicitly given in the ED guidance document, a structured scheme for evaluating each line of evidence was developed for the purpose of this study, including principles for categorizing lines of evidence as strong, moderate or weak. This step was considered necessary in order to conduct consistent WoE evaluation that could be compared between assessors, as well as communicated and justified to other stakeholders of the assessment. It should be noted that principles for categorizing lines of evidence have significant bearing on the conclusions of an assessment as they determine the terminology as well as basis for concluding whether evidence is strong, moderate or weak. In this case, we for example decided that a strong line of evidence would entail that “Effects were observed in one or more studies of high reliability; there are no conflicting results” (Table 2). One justified concern related to this principle could be that more than one study would be needed to reach the conclusion of “strong” evidence, since conflicting results are not possible when only one study is considered. However, this principle was formulated because in the regulatory setting it is not uncommon that only one study is providing data for a specific line of evidence. In our opinion, if there is data from a single reliable study showing an effect in an important parameter it should be possible to consider that evidence as strong. It should also be noted that the principles formulated here describe the evidence for effects observed, not absence of effect. In other words, it is not possible to apply these principles to conclude that there is strong, moderate or weak evidence for absence of effect. Similar to other steps of the WoE assessment process, for example the organization of data into different lines of evidence, setting up principles for WoE categorization is guided by expert judgment and will likely differ between assessors. It is therefore most important that such principles, when used, are clearly described. Efforts to further standardize and provide more detailed guidance for the WoE evaluation approach would contribute to the harmonization of the ED assessment process between evaluators, as well as between regulatory frameworks.

The approach for identifying EDs under the PPP and BP regulations does not allow for distinguishing between known and suspected EDs. While this distinction may not be directly necessary for the regulation of pesticides, it is an issue that becomes relevant for chemicals regulation in the context of other EU legislation. Under REACH, EDs are considered as of equivalent concern to substances classified as CMR substances according to the CLP regulation [47]. The European Parliament has also stated that EDs should be considered as such when developing a harmonized European framework for EDs [26]. Since CMR substances are classified as known, presumed or suspected as causing cancer, mutations or reproductive toxicity, EDs should be classified according to the same principles. The need for such an approach is also addressed in the European Commission’s Chemicals Strategy [4]. This issue was partly addressed in a previous proposal for a framework for the systematic review and integrated assessment (SYRINA) to identify EDs [35]. In this framework, an approach for classifying substances as known, probable or possible EDs was presented. Although deviating somewhat from the process for identifying EDs described in the ED guidance document, the main principles for identifying an ED based on the WHO definition, as well as systematic review methodology, are the same in the SYRINA framework.

## Conclusions

The present study illustrates the application of the EU criteria and guidance in the assessment of ED properties of a REACH chemical. In this case, the available information was sufficient to complete all the steps of the process for ED identification as set out in the ED guidance document, and to identify ED properties of the model substance BPAF. However, the large amount of toxicological information needed for ED assessment raises concerns, especially in relation to the relatively limited regulatory information requirements for chemicals regulated under REACH. It is likely that the data will often be insufficient to draw conclusions about both EATS-mediated adversity and endocrine activity. This case study especially highlights the importance of mechanistic understanding and data in the identification of ED properties among chemicals. The current rapid development of novel in vitro and in silico methods is promising and can significantly contribute to fill information gaps regarding different EATS and non-EATS mechanisms and lead to increased confidence in identifying EDs. However, the reliability and regulatory relevance of such methods need to be ensured, which requires joint efforts and collaborations between method developers, researchers and regulatory authorities.

Many endocrine-mediated effects are not specifically investigated in standardized tests in the current OECD conceptual framework for ED testing. Current developments are on-going and the ED guidance document anticipates the development of new OECD-approved test methods that include evaluation of endpoints relevant for endocrine disruption, such as non-EATS mediated effects. However, it is crucial to acknowledge that currently the identification and evaluation of both ED-related adversity and endocrine activity are heavily reliant on expert judgment. This puts high demands on promoting structure and transparency in the process for identifying and assessing EDs. This work identifies some points in the process, and especially in the ED guidance document, that can be further developed to improve structure and transparency.

Importantly, the current approach for identifying EDs under the PPP and BP regulations does not allow for distinguishing between known and suspected EDs in line with the classification of CMRs. This remains one main challenge for a harmonized EU approach to the identification and regulation of EDs.

## Supplementary Information

**Additional file 1: Table S1.** PECO statements defined for the present study. **Table S2.** Eligibility criteria stablished for research articles inclusion or exclusion. **Table S3**: Search terms, data search and number of items retrieved in the BPAF single target search for each database. **Table S4**: Number of references and date of BPAF search in the three databases and applicants dossier.

**Additional file 2.** 88 studies.

**Additional file 3.** Lines of Evidence.

## Data Availability

Not applicable.
