# Supplementary material for: Assessment of the endocrine disrupting properties of bisphenol AF: a case study applying the European regulatory criteria and guidance
Source: Environ Health. 2021 Apr 24;20:48. doi: 10.1186/s12940-021-00731-0 (PMC8070297; doi:10.1186/s12940-021-00731-0)
Supplement: Supplementary file 1 — Additional file 1: Table S1. PECO statements defined for the present study. Table S2. Eligibility criteria stablished for research articles inclusion or exclusion. Table S3: Search terms, data search and number of items retrieved in the BPAF single target search for each database. Table S4: Number of references and date of BPAF search in the three databases and applicants dossier. [file 12940_2021_731_MOESM1_ESM.docx]

**Table S1.** PECO statements defined for the present study.

| **PECO statements** | |
| --- | --- |
| **Population** | *In vivo* (mammalian, amphibian, fish), Mechanistic (*in vitro*, animal/human cell lines, animal/human tissue models, primary cells, organ cultures), Human (epidemiology). |
| **Exposure** | Bisphenol AF |
| **Comparator** | Control versus exposed in experimental settings.  Different dose levels (no restriction regarding different life stages/sex/generations). |
| **Outcome** | Estrogen, Androgen, Thyroid and Steroidogenesis-related endpoints.  (any EATS-related outcomes indicative of adversity and/or endocrine activity). |

**Table S2.** Eligibility criteria stablished for research articles inclusion or exclusion.

| **Eligibility (inclusion/exclusion) criteria** | |
| --- | --- |
| **Inclusion criteria** | Contain the aspects set out in the PECO statements. |
| **Exclusion criteria** | Ineligible exposure (e.g. mixture exposure).  No original data (e.g. review article).  Full-text not available.  Ecotoxicology studies (algae, invertebrates, field studies)  Environmental studies not assessing effects (eg. environmental faith, prevalence in soil, foods…). |

**Table S3**: Search terms, data search and number of items retrieved in the BPAF single target search for each database

| Database | Search terms with boolean operators and limitations (a) | Data of search | Number of items |
| --- | --- | --- | --- |
| Web of Science | ("Bisphenol AF" or BPAF or BIS-AF or "1478-61-1" or "Bisphenol AF-M" or "2,3,5,6-tetrafluoro-4-[1-fluoro-2-(4-hydroxyphenyl)propan-2-yl]phenyl] hypofluorite" or "4,4'-(1,1,1,3,3,3-hexafluoro-2,2-propanediyl)diphenol" or "hexafluoroacetone bisphenol" or "hexafluoroacetone bisphenol A" or "hexafluorobisphenol A" or "hexafluorodiphenylolpropane" or "hexafluoroisopropylidenebis(4-hydroxybenzene)" or "phenol, 4,4'-[2,2,2-trifluoro-1-(trifluoromethyl)ethylidene]bis-" or "phenol, 4,4´-[2,2,2-trifluoro-1-(trifluoromethyl)ethylidene]bis- (9CI)" or "phenol, 4,4´-[2,2,2-trifluoro-1-(trifluoromethyl)ethylidene]di- (6CI, 7CI)" or "phenol, 4,4´-[trifluoro-1-(trifluoromethyl)ethylidene]di- (8CI)" or "1,1,1,3,3,3-hexafluoro-2,2-bis(4-hydroxyphenyl)propane" or "2,2-bis(4´-hydroxyphenyl)hexafluoropropane" or "2,2-bis(4-hydroxyphenyl)-1,1,1,3,3,3-hexafluoropropane" or "2,2-bis(4-hydroxyphenyl)hexafluoropropane" or "2,2-bis(4-hydroxyphenyl)perfluoropropane" or "2,2-bis(p-hydroxyphenyl)hexafluoropropane" or "4,4'-(1,1,1,3,3,3-hexafluor-2,2-propandiyl)diphenol" or "4,4'-(1,1,1,3,3,3-hexafluoro-2,2-propanediyl)diphénol" or "4,4'-(1,1,1,3,3,3-hexafluoropropane-2,2-diyl)diphenol” or "4,4'-(2,2,2-trifluoro-1-(trifluoromethyl)ethylidene)bisphenol" or "4,4'-(2,2,2-trifluoro-1-(trifluoromethyl)ethylidene)diphenol" or "4,4'-(hexafluoroisopropylidene)diphenol" or "4,4'-[2,2,2-trifluoro-1-(trifluoromethyl)ethylidene]diphenol" or"4,4´-(hexafluoroisopropylidene)diphenol" or "4,4´-[2,2,2-trifluoro-1-(trifluoromethyl)ethylidene]bisphenol" or "4,4´-[trifluoro-1-(trifluoromethyl)ethylidene]diphenol" or "4,4′-(hexafluoroisopropylidene)diphenol" or "4-[1,1,1,3,3,3-hexafluoro-2-(4-hydroxyphenyl)propan-2-yl]phenol") | 15/02/2019 | 446 |
| PubMed | ("4,4'-hexafluorisopropylidene diphenol"[Supplementary Concept] or Bisphenol AF or BPAF or BIS-AF or "2,2-bis(4-hydroxyphenyl)hexafluoropropane" or "4,4'-(hexafluoroisopropylidene)diphenol" or "4,4´-(hexafluoroisopropylidene)diphenol") | 15/02/2019 | 168 |
| Embase | ('bisphenol af'/exp or 'Bisphenol AF' or BPAF or ('4,4′-(Hexafluoroisopropylidene)diphenol'):ti,ab,kw | 12/04/2019 | 225 |

(a): Search terms, boolean operators and limitations are adapted to each database requirements

**Table S4**: Number of references and date of BPAF search in the three databases and applicants dossier

| **Database** | **Number of references** | **Date of search** |
| --- | --- | --- |
| Web of Science | 446 | 15^th^ February 2019 |
| Pubmed | 168 | 15^th^ February 2019 |
| Embase | 225 | 12^th^ April 2019 |
| Total number | 839 |  |
| After deduplication | 511 |  |
